# Analysis of Patients' Online Reviews of Orthopaedic Surgeons

**DOI:** 10.5435/JAAOSGlobal-D-22-00074

**Published:** 2022-10-18

**Authors:** Ellis M. Berns, Daniel B. C. Reid, George M. Anderson, Daniel Alsoof, Benjamin Shapiro, Andrew S. Zhang, Alan H. Daniels

**Affiliations:** From the Warren Alpert Medical School of Brown University, Providence, RI (Berns and Anderson); the San Diego Spine Foundation, San Diego, CA (Dr. Reid); the Department of Orthopaedic Surgery, Warren Alpert Medical School of Brown University, Providence, RI (Dr. Alsoof, Dr. Zhang, and Dr. Daniels); and the Department of Psychiatry , Dartmouth-Hitchcock Medical Center, Lebanon, NH (Dr. Shapiro).

## Abstract

Physician rating websites (PRWs) are an increasingly popular interface between patient and surgeon. Despite the growing popularity of PRWs, little guidance exists for orthopaedic surgeons regarding online reviews. We analyzed online ratings and comments to provide a better understanding of patients' values and expectations so that surgeons can tailor their practice accordingly to enhance their clinical care and online reputation.

**Methods:**

Three common PRWs (Vitals, HealthGrades, and RateMDs) were queried from January 1, 2006, to May 18, 2020. Publicly available ratings, both quantitative (1 to 5 stars) and qualitative (free text comments), were collected. Comments were qualitatively tabulated as having positive or negative assessments for categories including outcome, personality, staff, surgical skill, visit time, bedside manner, wait time, diagnosis, knowledge, treatment, and advanced practice providers and analyzed using chi square goodness of fit. Quantitative comparisons of star ratings were made across surgeon years in practice, sex, practice setting, and PRW and compared using chi square independence testing.

**Results:**

In total, 81% of patient comments were found to have a positive assessment. Comments regarding outcome (*P* < 0.001), staff (*P* = 0.001), surgical skill (*P* < 0.001), or knowledge (*P* = 0.001) were more likely to be positive. Reviews regarding bedside manner (*P* < 0.001), wait time (*P* < 0.001), diagnosis (*P* < 0.001), treatment (*P* < 0.001), or advanced practice providers (*P* < 0.001) were more likely to be negative. Surgeon sex was not associated with a difference in quantitative ratings (*P* = 0.131), unlike practice setting (*P* < 0.001) and PRW (*P* < 0.001).

**Discussion:**

PRWs are a growing interface between surgeon and patient with a considerable effect on surgeon marketability. This study reveals a statistical association between certain patient-centered medical practices and positive patient reviews. This emphasizes the importance of ensuring that high standards are maintained throughout a physician's practice of maintaining a constant awareness of the fundamentals for effective patient care and of taking care to curate a physician's online presence.

Physician rating websites (PRWs) have become increasingly popular since the advent of the digital age.^1^ Studies have shown that up to 25% of patients visit PRWs and that physician rating influences the choice of physician.^2,3^ The content of online reviews are varied and may include clinical outcomes, physician-patient relationship, perception of office staff, wait times, and cost of health care.^4,5^ Orthopaedic surgeons, specifically, are often rated not on their surgical ability or outcomes but on their perceived bedside manner and availability.^5^ Many PRWs do not require patient verification to submit comments and do not have standardized criteria, leading to concerns about their accuracy and authenticity.^5,6^ Previous studies have shown that younger surgeons and academic practices are more likely to receive higher ratings.^7,8^ In a national study, Frost and Mesfin^9^ found that 94.3% of orthopaedic surgeons were rated on at least one PRW with an average score of 71.4 of 100.

The existing literature has characterized online ratings across orthopaedic subspecialties including general practice, spine, hand, sports medicine, and foot and ankle;^7,8,10–12^ however, to the best of our knowledge, no study has previously analyzed patient's free written reviews across all orthopaedic specialties.

Despite the apparent importance of PRWs, there are few recommendations for orthopaedic surgeons to navigate this challenging online landscape. The primary goal of this study was to review common online PRWs in an effort to better understand what measures are deemed important to patients so that surgeons may tailor their practices and behaviors accordingly. We also examined trends for positive and negative online reviews. An improved understanding of patients' values, preferences, and needs may help surgeons provide more patient-centered care, enhancing both the clinical care of the patient and the online reputation of the surgeon.

## Methods

Three common PRWs (Vitals, HealthGrades, and RateMDs) were queried for this investigation. All actively practicing orthopaedic surgeons in the state of Rhode Island were included in this study. The state of Rhode Island was chosen as a study location because of its balance of a major academic hospital and multiple private practices to capture a diverse yet contained patient and surgeon population. Surgeon's practices were categorized as either academic or private based on whether the practice was associated with a major academic health institution. This information was collected from publicly available online search engines.

Data extraction was completed in May 2020. Ratings from the earliest available rating (January 1, 2006) to present (May 18, 2020) were used for this study. Publicly available quantitative (1 to 5 stars) and qualitative (free form) reviews were collected for all available online data up to the point of extraction, resulting in reviews for examination from 2006 to 2020. The start of this period was chosen because of the first available reviews on the selected PRWs for our cohort of surgeons, and no reviews were excluded. Qualitative ratings were tabulated based on “stars,” reported as integers from 1 to 5. Free-text comments, if present, were read and qualitatively tabulated as being positive or negative assessments for specific categories including outcome, personality, staff, surgical skill, visit time, bedside manner, pain, wait time, diagnosis, knowledge, treatment, accessibility, office, advanced practice providers (APPs), financial or insurance, and workers' compensation. Free-text comments contained assessments on up to five categories, including a possible mix of positive and negative assessments. Reviews with unclear comments were designated as “unclear” and not included in analysis.

The first author conducted the initial primary review of the free-text comments, with the senior author providing a secondary review of all data and tabulations. For the final assessment, all authors reviewed the free-text comments. Disagreements were rectified by a majority vote by all authors on this study. Surgeon demographic data included surgeon sex, practice setting, and years in practice. Years in practice was determined based on fellowship completion date (or residency completion date in the case of orthopaedic generalists), which were collected from publicly available online search engines.

SPSS Statistics 27 for Mac (IBM) was used for statistical analysis. Significance was set at *P*-value < 0.05 a priori. Chi square goodness-of-fit tests were used to compare assessment with each category to the overall percentage of positive and negative assessments. Chi square tests of independence with Cramer V were run to test the number of ratings between sex, practice setting, PRW. Kruskal-Wallis testing with post hoc analysis was used to compare years in practice.

## Results

**Figure 1:**
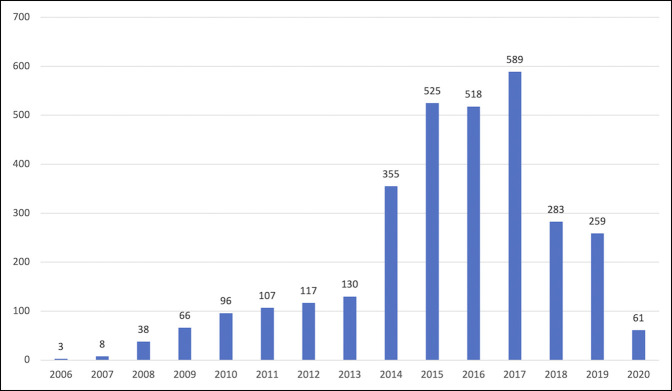
Graph showing the number of reviews over time.

A total of 3155 reviews were examined, of which 1714 included free-text comments. Across all categories, the comments contained 2427 positive and 554 negative assessments (81.4% positive patient comments). The number of reviews across all platforms was lowest in 2006 with three reviews and highest in 2017 with 589 reviews (Figure 1).

**Figure 2:**
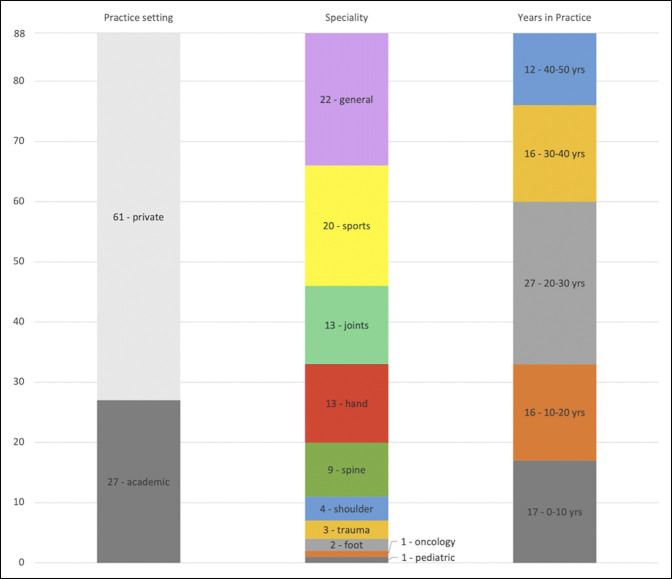
Graph showing characterization of surgeons in this study.

In total, 88 orthopaedic surgeons from 10 different subspecialties were reviewed. Overall, 27 physicians were in academic practice and 61 were in private practice. Seventeen physicians had been in practice for 0 to 10 years; 16 physicians had been in practice for 10 to 20 years; 27 physicians had been in practice for 20 to 30 years; 16 physicians had been in practice for 30 to 40 years; and 12 physicians had been in practice for 40 to 50 years (Table 1 and Figure 2).

**Table 1:** Number of Reviews by Star Rating (1 to 5) Across Surgeon Sex, Practice Setting, and Physician Rating Websites (PRWs) With Chi Square Independence Testing Results

	1	2	3	4	5	Total	χ^2^ *P* value	Cramer V
Sex								
M	506	78	140	271	2088	3083	0.131	0.047
F	10	5	1	6	50	72		
Setting								
Academic	114	28	43	74	972	1231	<0.001	0.197
Private	402	55	98	203	1166	1924		
PRW								
Vitals	333	50	119	197	1471	2170	<0.001	0.166
HealthGrades	122	6	4	12	488	632		
RateMDs	61	27	118	68	179	353		

**Figure 3:**
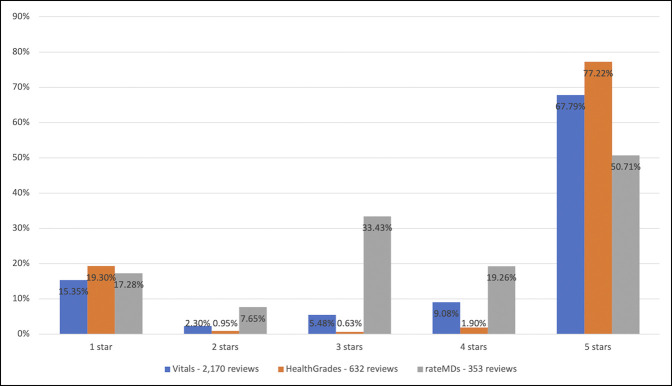
Graph comparing online platforms for the number of star ratings.

**Figure 4:**
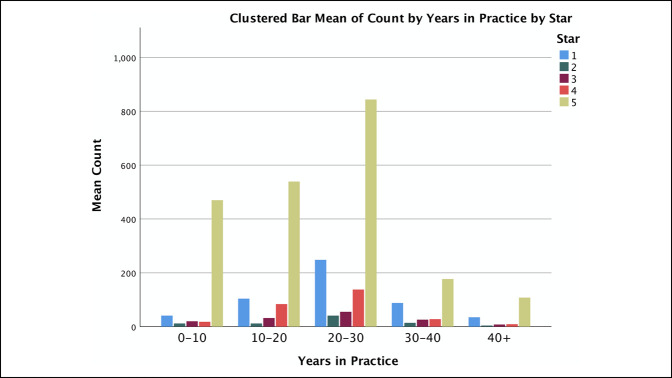
Graph showing the count of star ratings by surgeon's years in practice.

Surgeon sex was not associated with a difference in star ratings with male surgeons receiving 68% five-star ratings and female surgeons receiving 69% five-star ratings (*P* = 0.131). A statistically significant difference was observed between surgeons who practice in an academic setting, with 78% five-star ratings, and a private setting, with 61% five-star ratings (*P* < 0.001). A statistically significant difference was also observed between PRWs, with Vitals found to have 68% five-star ratings, HealthGrades with 77% five-star ratings, and RateMDs with 51% five-star ratings (*P* < 0.001) (Table 2 and Figure 3). Surgeons 0 to 10 years in practice had 82% five-star ratings, significantly higher compared with each other range for years in practice (*P* < 0.001). Surgeons 10 to 20 years in practice had 70% five-star ratings, markedly higher compared with those with 20 to 30 or 30 to 40 years of practice (*P* < 0.001) (Figure 4).

**Table 2:** Positive and Negative Assessments by Patients' Experience Subcategory Discussed in Physician Rating Websites (PRWs) Free-Text Comments With Chi Square Goodness-of-Fit Testing

	Positive	Negative	χ^2^ *P* value
Outcome	632	52	<0.001^a^
Personality	540	138	0.236
Staff	308	41	0.001^a^
Surgical skill	193	4	<0.001^a^
Visit time	165	30	0.251
Bedside manner	110	58	<0.001^a^
Pain	120	19	0.136
Wait time	65	69	<0.001^a^
Diagnosis	72	46	<0.001^a^
Knowledge	100	7	0.001^a^
Treatment	31	36	<0.001^a^
Accessibility	36	5	0.293
Office	34	5	0.355
Advanced practice providers (APPs)	16	17	<0.001^a^
Financial or insurance	1	21	0.082
Workers' compensation	4	6	0.090
Total	2427	554	

^a^Statistically significant result.

In total, 81% of patient comments were deemed positive. Comments that mentioned outcome (*P* < 0.001), staff (*P* = 0.001), surgical skill (*P* < 0.001), and knowledge (*P* = 0.001) were significantly more likely to have a positive assessment than typical comments. Comments that mentioned bedside manner (*P* < 0.001), wait time (*P* < 0.001), diagnosis (*P* < 0.001), treatment (*P* < 0.001), and APPs (*P* < 0.001) were more likely to have a negative assessment. Comments mentioning personality (*P* = 0.236), visit time (*P* = 0.251), pain (*P* = 0.136), physician accessibility (*P* = 0.293), office (*P* = 0.355), finance or insurance (*P* = 0.082), or workers' compensation (*P* = 0.090) were not significantly more likely to have a positive or negative assessment than the typical comments. These results are summarized in Table 3.

**Table 3:** Summary of Recommendations to Improve Ratings With Appropriate Evidence From Free-Text Analysis of Patient Physician Rating Website (PRW) Comments

Recommendations	Evidence
• Surgeons should suggest patients with positive surgical outcomes to leave reviews on PRWs at an opportune time such as a postoperative visit	• Patients with good surgical outcomes and who perceived their surgeon as skilled were more likely to leave positive reviews (*P* < 0.001 for each)
• Surgeons should prioritize their bedside manner during every patient interaction by focusing on kindness, professionalism, and honesty	• Patients were more likely to comment negatively on surgeon bedside manner (*P* < 0.001) while being neutral about physician personality (*P* = 0.236)
• To avoid patients waiting for their visit, surgeons should stay within scheduled visit times and consider more focused patient visits that take up less time	• Patients were neutral about visit time (*P* = 0.251) while more likely to negatively comment on wait time for a visit (*P* < 0.001)
• Less invasive treatments should be considered before surgical intervention, except in the case when the patient clearly desires surgery	• Patients were more likely to comment negatively on treatment when they felt like they were rushed into surgery (*P* < 0.001).
• Patients who specifically request to see the surgeon instead of an advanced practice provider (APP) should be scheduled with the surgeon	• Patients were more likely to comment negatively when seeing an APP instead of a surgeon (*P* < 0.001).

## Discussion

Our results indicate that patients who have a perceived positive outcome are more likely to comment positively on PRWs. Thus, physicians seeking to improve their online reputation should focus first on achieving good outcomes for their patients. Furthermore, surgeons may consider encouraging postoperative patients with perceived positive outcomes to fill out online reviews. Concordantly with their outcome, patients who perceived their surgeon as skilled were more likely to leave positive reviews. Patients were also more likely to comment positively on staff interaction. Although not directly a metric of the surgeon, these findings should encourage surgeons to establish professional and efficient workplace environments which facilitate improved patient-centric care through team-based approaches. Furthermore, positive comments about treatment included the patient not feeling rushed into surgery, a finding in line with common knowledge that we found supported by statistical significance.

Regarding negative ratings, patients were more likely to comment negatively on bedside manner while comments on surgeon personality were not markedly positive or negative. Despite the inherent overlap in these categories, bedside manner was measured when a comment specifically used that term, rather than describing a positive personality trait, such as kindness, professionalism, or attentiveness, or a negative personality trait, such as rudeness or uncaring. These findings corroborate those of Bakhsh et al.^12^ who also noted that bedside manner, knowledge, and wait time influenced a surgeon's rating. Similarly, Velasco et al.^10^ found that, among orthopaedic foot and ankle surgeons, positive comments were written about physician personality and communication while negative comments addressed bedside manner and waiting time. With multiple sources now demonstrating that poor bedside manner is likely to negatively influence online reputation, surgeons should work to improve their bedside manner, both to improve patient care and to enhance PRW ratings.

Wait time, incorrect diagnosis, incorrect treatment, and APPs were more likely to result in negative PRW reviews. To minimize wait times, surgeons should be conscious of overbooking and not exceeding predetermined appointment times. Patient visit time did not affect online reviews either positively or negatively (*P* = 0.260). Therefore, surgeons should consider focused visits for simple problems, postoperative visits, and follow-ups to maintain a well-defined and efficient schedule. For complex patient visits which may require more extensive history-taking, physical examination, discussion, or decision making to provide appropriate care, surgeons should consider scheduling longer visit times to avoid falling behind schedule. For diagnosis, patients often commented that their surgeon corrected a prior misdiagnosis or were seen as second opinions. Special consideration should be taken for patients who may not agree with the direction another surgeon has given them. Patients often commented negatively that they were unable to see the doctor and instead saw an APP. Although the incorporation of APPs into surgical practices may improve practice efficiency and expand access to care, surgeons should be careful to avoid using APPs for important clinical encounters including complex diagnosis and preoperative decision making. Many patients desire to discuss such important topics with their surgeon specifically. In cases where patients specifically request direct access to their surgeon, every attempt should be made to schedule accordingly because this is likely to improve patient satisfaction and avoid negative PRW reviews. A summary of evidence-based recommendations for surgeons to improve their online PRW reputation is given in Table 3.

It should be noted that several categories had more positive comments than negative ones, such as physician personality, visit time, pain control, and accessibility. Although these categories were not markedly more positive or more negative compared with the typical comments (81% positivity), they reinforce the importance of maintaining these qualities. Furthermore, categories such as bedside manner and diagnosis were mostly positive; however, compared with the typical comments, they were markedly more negative.

To help manage their online presence, surgeons can register and “claim” their profile on PRWs. HealthGrades, Vitals, and RateMDs allow for surgeons or an authorized representative to manage their online profile. One administrative account can manage multiple surgeons' profiles at once. Frequent review of online comments for feedback and rapid addressing of negative reviews may be beneficial. Surgeons can compile prewritten responses for common negative reports, such as wait time or bedside manner, which can then be tailored to the individual patient's comment. This response could include a means to contact the surgeon or an administrative representative who could discuss the issue directly with the patient. If the patient thinks that their concern has been addressed, they have the option to modify their original review. Furthermore, although receiving a bad review may be discouraging, surgeons should be aware that their effect can be diluted by receiving many good reviews. Surgeons can ask patients who were happy with their care to leave a review.

In our study, academic orthopaedic surgeons were more likely to receive higher reviews than those in private practice. Patients were also more likely to leave positive reviews concerning their perception of a surgeon's knowledge. Although not explicitly detailed in the PRWs, patient perception of a surgeon's knowledge is likely predicated on a combination of online reputation, word-of-mouth reputation, academic productivity, academic associations, and clinical explanation of diagnosis and treatment. Haglin et al.^13^ found that in the Northeast, spine surgeons with recent publications were more likely to be perceived as more trustworthy by patients, suggesting that academic physicians may rate higher on PRWs. Frost and Mesfin^9^ also found that academic practices were associated with higher ratings.

Surgeons 0 to 10 years in practice had markedly higher ratings, a result consistent with previous studies. Runge et al. measured arthroplasty surgeon reviews and found that surgeons in practice for 1 to 10 years had markedly higher ratings than those in practice for 11 to 20 and 21+ years. They did not find a difference between sex, practice type, and geographic region.^14^ Frost and Mesfin^9^ noted that physicians 6 to 10 years in practice were associated with higher ratings compared with physicians who had been in practice longer. We hypothesize that surgeons starting practice are more likely to have a discrete digital strategy for online reputation management than surgeons with more established careers and that such a discrete focus may be related to the higher average ratings. Younger surgeons may feel more comfortable navigating this digital space and may ask their patients to leave reviews. In addition, it is possible that younger patients feel more comfortable using PRWs and may identify more with younger surgeons. Older patients may not feel comfortable writing digital reviews and, therefore, may benefit from education on the use of PRWs. This would provide better feedback to represent this age group. Furthermore, these findings suggest that more experienced surgeons may especially benefit from PRW analysis and adjusting their practices and/or digital reputation management strategy accordingly.

Most online physician reviews use a breakdown of factors to calculate their overall score and provide a text box for freehand comments.^12^ We used a similar method to the study by Velasco et al.^10^ by breaking down common themes in patient free-text responses instead of the structured survey provided by some PRWs. A recent review of studies examining orthopaedic PRWs by Bernstein et al. found that younger age, social media presence, trustworthiness, time with patients, and answering questions were associated with higher ratings.^15^ We analyzed PRW free-text comments to provide a measurement of qualities that influence a patient's interaction with their orthopaedic surgeon and likewise their responses on PRWs.

Although PRWs are an imperfect system, they are likely to influence patient preference for the foreseeable future. There are several factors which are under the surgeon's direct control which influence ratings, including on-time clinical visits, patient outcomes, and bedside manner. Furthermore, surgeons have secondary influence on other factors, such as the friendliness of clinical and nonclinical staff and clinic efficiency.

This investigation has several potential limitations. The subjective interpretation of patient comments may have obscured true patients' perspectives on their surgeons. Furthermore, there is subjectivity in identifying why patients may choose to leave a review online, and this cannot be taken into consideration within the analysis. However, all reviewers were captured for this time frame at this location, and care was taken to ensure standardization by using definitions for category inclusion. All authors additionally reviewed each comment for categorization to help provide a more generalizable analysis of the data. Another limitation is the lack of demographic information on the reviewers themselves. It is likely that patients who submit online reviews do not represent the entire patient population. Despite these limitations, these findings have clinical significance because they demonstrate what factors are associated with a positive patient experience. The results of our analysis can inform clinical practice, which are summarized by the recommendations in Table 3. This includes critical components of good patient care including timely consultation, scheduling requests, and bedside manner.

## Conclusion

PRWs can have an immense effect on surgeon marketability and overall image to the public. These websites are largely unregulated and serve as a common interface between surgeons and their patients. This study reveals a statistical association between certain patient-centered medical practices and positive patient reviews. This emphasizes the importance of ensuring that high standards are maintained throughout a physician's practice and a constant awareness of the fundamentals for effective patient care.
